# High-resolution mapping of reproductive tract infections among women of childbearing age in Bangladesh: a spatial-temporal analysis of the demographic and health survey

**DOI:** 10.1186/s12889-021-10360-4

**Published:** 2021-02-12

**Authors:** Chenyang Feng, Ruixue Li, Abu Ahmed Shamim, Md Barkat Ullah, Mengjie Li, Rubee Dev, Yijing Wang, Tingting Zhao, Jing Liao, Zhicheng Du, Yuheng Ling, Yingsi Lai, Yuantao Hao

**Affiliations:** 1 Department of Medical Statistics, School of Public Health, Sun Yat-Sen University, Guangzhou, China; 2 Department of Information, Sun Yat-Sen University Cancer Center, Guangzhou, 510060 China; 3 James P Grant School of public Health, BRAC University, Dhaka, Bangladesh; 4 Department of Nutrition, University of California Davis, California, USA; 5 Dhulikhel Hospital, Kathmandu University Hospital, Kavre, Nepal; 6 Sun Yat-sen Global Health Institute, Sun Yat-Sen University, Guangzhou, China; 7 CNRS UMR 6240, Universite de Corse Pascal Paoli, 20250 Corti, France

**Keywords:** Reproductive tract infections, Spatial-temporal analysis, High-resolution map, Bangladesh

## Abstract

**Background:**

Reproductive tract infections (RTIs) have become major but silent public health problems devastating women’s lives in Bangladesh. Accurately and precisely identifying high-risk areas of RTIs through high-resolution risk maps is meaningful for resource-limited settings.

**Methods:**

We obtained data reported with RTI symptoms by women of childbearing age in the years 2007, 2011 and 2014 from Bangladesh Demographic and Health Survey. High-spatial Environmental, socio-economic and demographic layers were downloaded from different open-access data sources. We applied Bayesian spatial-temporal models to identify important influencing factors and to estimate the infection risk at 5 km spatial resolution across survey years in Bangladesh.

**Results:**

We estimated that in Bangladesh, there were approximate 11.1% (95% Bayesian credible interval, BCI: 10.5–11.7%), 13.9% (95% BCI: 13.3–14.5%) and 13.4% (95% BCI: 12.8–14.0%) of women of childbearing age reported with RTI symptoms in 2007, 2011 and 2014, respectively. The risk of most areas shows an obvious increase from 2007 to 2011, then became stable between 2011 and 2014. High risk areas were identified in the southern coastal areas, the western Rajshahi Division, the middle of Khulna Division, and the southwestern Chittagong Division in 2014. The prevalence of Rajshahi and Nawabganj District were increasing during all the survey years.

**Conclusion:**

The high-resolution risk maps of RTIs we produced can guide the control strategies targeted to priority areas cost-effectively. More than one eighth of women of childbearing age reported symptoms suggesting RTIs and the risk of RTIs varies in different geographical area, urging the government to pay more attention to the worrying situation of female RTIs in the country.

**Supplementary Information:**

The online version contains supplementary material available at 10.1186/s12889-021-10360-4.

## Background

Sexual and reproductive health and rights, currently featured on the Sustainable Development Goal agenda, are fundamental for sustainable development [1, 2]. Female reproductive tract infections (RTIs), playing an important role on reproductive health, become a major public health concern, particularly in developing countries where they are endemic [3]. Women RTIs refer to endogenous, iatrogenic or sexually transmitted infections affecting the reproductive tract of women. They usually originate in the lower reproductive tract (e.g., vaginitis or cervicitis) and may produce symptoms such as abnormal vaginal discharge, genital pain, itching and burning feeling with urination [4–6]. Women of childbearing age, generally referring to women aged 15–49 years old, are the most susceptible group to RTIs, as they are in child-bearing period and have frequent sexual life [7, 8]. In developing countries, RTIs become major but silent public health problems devastating women’s lives. More than 1 million women and infants die of the complications (e.g., postabortal and puerperal sepsis, ectopic pregnancy, fetal and perinatal death, and infertility) resulted from RTIs each year [9]. Besides, untreated infections increase the risk of other diseases, such as acquiring and transmitting human immunodeficiency virus (HIV) infection, chronic lower abdominal pain, emotional distress social rejection of women and cervical cancer [10].

Bangladesh, classified as the Least Developed Countries by United Nations, has high risk of poor female reproductive health [11], although the Government of Bangladesh has pursued a number of strategies, such as the Urban Primary Health Care Project, seeking to provide services to women of victims of violence and to respond to the reproductive [12] and Initiation of Family Planning Programme [13]. To our knowledge, there is no nationwide report on RTIs in Bangladesh currently, but related data from different surveys suggest a worrying situation. A survey in the capital city Dhaka, found the prevalence of bacterial vaginosis and candida reached 23.3 and 32.5%, respectively, based on 2579 randomly selected married women [14]. In high-risk groups such as sex workers, up to 67.4% of women have cervical and/or vaginal infection [15]. The prevalence of female RTIs varies across the country, thus understanding the spatial-temporal distribution of RTIs and the corresponding influencing factors will be of crucial importance for guiding intervention strategies in limited resource settings.

Risk maps have been widely used in epidemiological studies for describing disease risk distribution [16–18]. Bayesian geostatistical modeling is one of the most rigorous inferential approaches for reliable risk estimates. It explores the association of survey data with potential influencing factors, employs spatial-specific random effects accounting for other unknown or unobtainable factors, and estimates the disease risk at areas without observed data [19]. In other words, it estimates disease risk (dependent variable) at areas without observed disease data (e.g., areas lack of surveillance or areas un-sampled in surveys), based on known potential influencing predictors (independent variable) at the corresponding areas and the spatial correlations between areas with disease survey data and that without. High spatial resolution maps track the geospatially pattern of disease risk, underscore high risk areas [20], and help health workers and policy makers to precisely identify priority areas where effective interventions should be targeted. In resource-limited countries, such maps are especially meaningful for allocation of limited medical resources to where most needed in a cost-effective manner.

In this study, we aimed to apply geostatistical models to estimate the spatial-temporal distribution of RTIs at high spatial resolution in Bangladesh, based on both the survey data from Demographic and Health Survey (DHS) Programs in the years 2007, 2011 and 2014, and other datasets with environmental, climatic and socioeconomic information, providing policy makers with disease distribution data for this period, in order to inform interventions, and to provide a basis for future RTI research.

## Method

### Data

We obtained health related data of child-bearing age women in Bangladesh from three latest available Demographic and Health Survey (DHS) conducted in 2007, 2011 and 2014. DHS, began in 1984, are nationally-representative household surveys that provide data of a wide range of issues including birth rate, mortality, migration, family planning, maternal and child health, nutrition, family living conditions and education in developing countries [21]. Bangladesh DHS (BDHS) was conducted every three or 4 years from 1993 up to the present. The most recent survey was in 2017–2018, however, the corresponding data hasn’t been published yet. A two-stage cluster sampling design was used. Firstly, the country was divided into a number of enumeration areas (EAs) and a certain amount of EAs were sampled according to the proportion of urban and rural areas. EAs, known as survey clusters, are city blocks or apartment buildings in urban areas, or villages or groups of villages in rural areas, which cover an average of 100–120 households. Secondly, 30 randomly selected families from each cluster were surveyed, resulting in indicators not only nationally representative but representative at the lower level of DHS regions and urban/rural residence [22]. Family-, women- (age 15–49), and health-related information, such as house situation, reproductive history, marriage, were collected using standard questionnaires.

Coordinates of clusters were available as geo-located data. However, in order to maintain privacy of respondents, the geo-located data is displaced, which leaves the coordinates of all cluster containing random errors: i) urban clusters contain between 0 and 2 km of error; ii) rural clusters contain 0 and 10 km of error [4]. These errors may result in misclassified assignments of predictor variables in geostatistical analysis. Nevertheless, according to the guidelines on the GPS data published by DHS, point extraction provided adequately unbiased estimates for most surface types other than highly non-smooth surface [22, 23]. We fitted simultaneous autoregressive regression models, proposed by the guidelines of DHS (see Additional file 1 for details), to test the smoothness of the predictor surfaces in our study.

In consistent with other researches, we defined a woman reported with RTI symptoms if she reported to have abnormal genital discharge or genital sore/ulcer during the last 12 months [4, 24]. This and the potential influencing data (i.e., wealth index of household, education years of women, education years of husbands, rate of births attended by a skilled provider, number of children in a household, coverage of improved toilet facilities) were extracted from DHS dataset at individual level. As the survey prior to 2007 only investigated women’s specific gynecological health problems during the 6 months preceding the survey, while the 2007 and subsequent survey focused on having abnormal genital discharge or genital sore/ulcer during the last 12 months, we just used survey data in the 2007, 2011 and 2014 in order to have a uniform definition of RTIs symptoms for the outcome variable.

Besides, we introduced a suite of environmental, climatic, socioeconomic and demographic data with high spatial resolution, such as human influence index (HII) and elevation. They are often related to the social, health, accessibility and demographic factors that underlie geographic change [25], assist in explaining the spatial variation of outcome variables, and are committed to the accuracy of predictions. A detailed information of the data sources, data periods, temporal and spatial resolutions are listed in Table S1 in Additional file 1.

### Data processing

We summarized the DHS individual-level data to cluster-level (i.e., EAs) according to the same coordinates. Environmental, climatic and socioeconomic data at the survey clusters were extracted using ‘raster’ package in R (version 3.5.0). Continuous data was standardized to mean zero and standard deviation one. To avoid collinearity in the subsequent modeling analysis, we calculated the Pearson’s correlation coefficient between each pair of continuous variables. If the coefficient was greater than 0.8 in one pair of variables, the one among the pair, which was more meaningful or with better data quality was retained. .

The environmental and socioeconomic data obtained from data sources other than DHS database were at high spatial resolution (1 × 1 km^2^). Except data of water bodies, the data at pixels nearest to the clusters were extracted for the corresponding clusters. For the variable “distance to the nearest fresh water body”, we calculated the distance directly between clusters to water bodies. For the MODIS/Terra data, time resolution is high. We averaged the data each year and extracted the ones of survey years to the corresponding clusters.

### Model fitting and validation

Bayesian geostatistical logistic regression models were applied to obtain spatially explicit RTI risk estimates. We denoted *p*_*it*_ and *n*_*it*_ the probability of reporting with RTI symptoms and the number of total surveyed individuals at location *i* (*i* = 1, 2,…, *L*) in the survey year *t*, respectively, For cluster *i* at survey time *t*, *n*_*it*_ independent individuals were randomly sampled from the population (the *n*_*it*_ is not vey huge in our study), and each of the individuals had a binary outcome. So, we assumed the number of positive individuals *Y*_*it*_ follows a binomial distribution, that is *Y*_*it*_~*Binomial*(*p*_*it*_, *n*_*it*_). The covariates were modelled with the logit link function as follows:

$$ logit\left({p}_{it}\right)={\beta}_0+{\boldsymbol{X}}_{it}^T\boldsymbol{\beta} +{\delta}_{it}+{\lambda}_i $$

Here *β*_0_ is the intercept, ${\boldsymbol{X}}_{it}^T$ is the vector of covariates for location *i* of the year *t*, and ***β*** the vector of the corresponding coefficients. *λ*_*i*_ indicates a location-specific exchangeable random effect assumed to follow a zero-mean normal distribution ${\lambda}_i\sim N\left(0,{\sigma}_{nonsp}^2\right)$. *δ*_*it*_ is a spatiotemporal effect term, assumed to follow a stationary spatiotemporal Gaussian process.

To avoid “big *n* problem” arising when working with the dense covariance matrix of a Gaussian field (GF), a discretely indexed spatial random process Gaussian Markov random field (GMRF) with a sparse precision matrix was used to approximate the continuous spatial process GF, by the computationally effective approach SPDE [26, 27]. A proper triangulated mesh is constructed over the study region representing the spatial domain. ***ξ =*** (***ξ***_**1**_, …, ***ξ***_***T***_)′ is denoted as the *T* × *G*-dimentional GMRF, where *T* is the total discrete time points and *G* the number of vertices of the mesh. The joint distribution of ***ξ*** is expressed as ***ξ~N***(**0**, ***Q***^***−*1**^) with ***Q = Q***_***s***_ ⨂ ***Q***_***t***_. ***Q***_***t***_ is the *T*-dimensional precision function for temporal effect, which is defined as either autoregressive process of order 1 (AR1) or exchangeable. ***Q***_***s***_ is the sparse precision matrix of the spatial GMRF, coming from the SPDE representation of the GF with a Matérn covariance function, constructed with spatial correlation *C*(*d*) = (*κd*)^*ν*^*K*_*ν*_(*κd*) and spatial variance ${\sigma}_{sp}^2=1/\left(4\pi {\kappa}^2{\tau}^2\right)$. Here *d* is the Euclidean distance between pair of locations, *κ* a scaling parameter, *υ* a smoothing parameter usually kept fixed, *τ* the precision parameter and *K*_*υ*_ the modified Bessel function of second kind with order *υ* (same value as the smoothing parameter). the spatial range is defined as $R=\sqrt{8\upsilon }/\kappa$, regarded as the distance with spatial correlation becoming negligible (< 0.1).

For a given time point *t*, we have ***ξ***_*t*_ **=** *ρ****ξ***_*t* − 1_ ***+ w***_*t*_ under AR1 temporal process, where ${\boldsymbol{\xi}}_1\sim \boldsymbol{N}\Big(\mathbf{0},{\boldsymbol{Q}}_{\boldsymbol{s}}^{-\mathbf{1}}/\left(1-{\rho}^2\right)$ and ***w***_*t*_ is assumed temporally independent with ${\boldsymbol{w}}_t\sim \boldsymbol{N}\left(\mathbf{0},{\boldsymbol{Q}}_{\boldsymbol{s}}^{-\mathbf{1}}\right)$ and $$ \mathrm{Cov}\left({w}_{it},{w}_{j{t}^{\prime }}\right)=\Big\{{\displaystyle \begin{array}{c}0\  if\ t\ne {t}^{\prime}\\ {}{\sigma}_{sp}^2\  if\ t={t}^{\prime}\end{array}} $$. By approximation of the GF with GMRF using SPED approach, for a given location *i* at time point *t*, the spatial random effect *δ*_*it*_ can be expressed as ${\delta}_{it}=\sum \limits_{g=1}^G{a}_{ig}{\xi}_{gt}$, where *a*_*ig*_ is the generic element of the sparse weight matrix A, which maps the GMRF ***ξ*** from the *G* triangulation vertices of the mesh to the observation locations [28]. As the spatial random effect of any observational survey location at any study time can be expressed by the GMRF ***ξ*** and the weight matrix A, it’s not necessary to require survey locations exactly the same during survey times.

We first defined ***Q***_***t***_ (the precision function for temporal effect) as AR1 process with autoregressive coefficient |*ρ*| < 1. To handle the current situation of irregularly spaced survey times (i.e., 2007, 2011 and 2014), we set equally spaced time knots (i.e., 2007, 2010.5, 2014) and built the GMRF on the knots, that is ***ξ =*** (***ξ***_***t =*** **2007**_, ***ξ***_***t =*** **2010.5**_, ***ξ***_***t =*** **2014**_)′. The latent field for survey year 2011 ***ξ***_***t =*** **2011**_ is approximated by the projection of ***ξ*** based on B-spline basis function of degree one [28]. In case of the 95% BCI of posterior distribution of *ρ* including zero, which suggests statistically non-significant time-dependence between survey years, we set ***Q***_***t***_ = ***I*** (unstructured unit matrix) for exchangeable time effect of the spatial-temporal process for the final model.

Furthermore, to avoid spatial confounding between spatially structured effects and fixed-effect covariates [29, 30], we restricted the spatial random effect to the orthogonal complement of the fixed effect covariates, by setting a constraint of ***Bξ***^′^ = **0**^′^, where ***B*** is the orthogonal matrix from the QR decomposition of the covariates matrix ***X*** [31]. Considering the identification issues between the main fixed effect and the random effects, we set a sum-to-zero constraint to the location-specific effect and an integrate-to-zero constraint to the spatial-temporal random effect [32].

We adopted Bayesian inferential framework to estimate the parameters as well as hyperparameters. We fitted the model under Bayesian inferential framework using the INLA package in R (version 3.5.0) with INLA-SPDE approach [28, 33]. We didn’t have much information to specify precise prior distributions for the parameters. To avoid subjectivity in the choice of priors and to keep inferences in a reasonable range, we used minimally informative priors. They were set as distributions with large variances, covering a wide range of reasonable values according to the characteristics of parameters, thus provide good representation of genuine ignorance about the parameters and do not affect strongly on the posteriors. Priors were set for parameters and hyper-parameters as following: *β*_0_, *β*~*N*(0, 1000), log((1 + *ρ*)/(1 − *ρ*))~*N*(0,0.15), log(*τ*)~*N*(0.378, 10) and log(*κ*)~*N*(−1.64,10) and $1/{\sigma}_{nonsp}^2\sim gamma\left(\mathrm{1,0.00005}\right)$. In addition, default setting for the smoothing parameter *υ* = 1 was adopted, which was used for many previous studies. To assess model sensitivity to different settings of *υ*, we also tried alternative values *υ* = 0.5.

In order to identify the best set of predictors, we carried out Bayesian variable selection. The following variables were considered for variable selection: education years of women, the proportion of birth attendance by skilled provider, number of children, the proportion of improve toilet, wealth, normalized difference vegetation index, land surface temperature (LST) in the daytime, LST at night, elevation, moisture, Human influence index urban extents, the distance the nearest fresh water body. Firstly, to identify the best functional form (i.e., linear of categorical) of continuous potential predictors, we converted continuous variables to three-level categorical ones according to preliminary exploratory graphical analysis. For each continuous potential predictor, we fitted two univariate Bayesian geostatistical models with the predictor as the only fixed effect independent variable, one with it in linear form and the other in categorical form. The deviance information criterion (DIC) and marginal predictive likelihood (MPL) were both recorded, and the functional form with smaller MPL and DIC of the model was selected.. Secondly, we fitted geostatistical models with all possible combinations of potential predictors as covariates and select those with smallest DIC and MPL as best set of predictors for the final model.

We carried out 10-fold cross-validation to assess the model performance. Survey locations were randomly divided 10 times in 90% (training set) and 10% (validation set) splits and the following performance indicators were calculated: (i) mean error (mean of observed prevalence minus predicted one), and (ii) the percentage of observations covered by 95% Bayesian credible intervals (BCI) of posterior predicted prevalence [17].

Estimates of RTI risk for each survey year were done using the INLA package over a grid of 7137 pixels across Bangladesh at 5 × 5 km spatial resolution. Pixel-level number of infected women was also calculated (Additional file 1). The results were mapped using ArcGIS 10.0. Furthermore, we calculated population-adjusted prevalence (median and 95%BCI) for all the 8 divisions (administrative divisions of level one, ADM1) and 64 districts (administrative divisions of level two, ADM2) in Bangladesh, by summarizing the pixel-level estimates to the corresponding ADM1 or ADM2 level. Risk changes over time at ADM2 level were mapped to show the temporal trend clearly.

## Result

### Data summaries

**Fig. 1:**
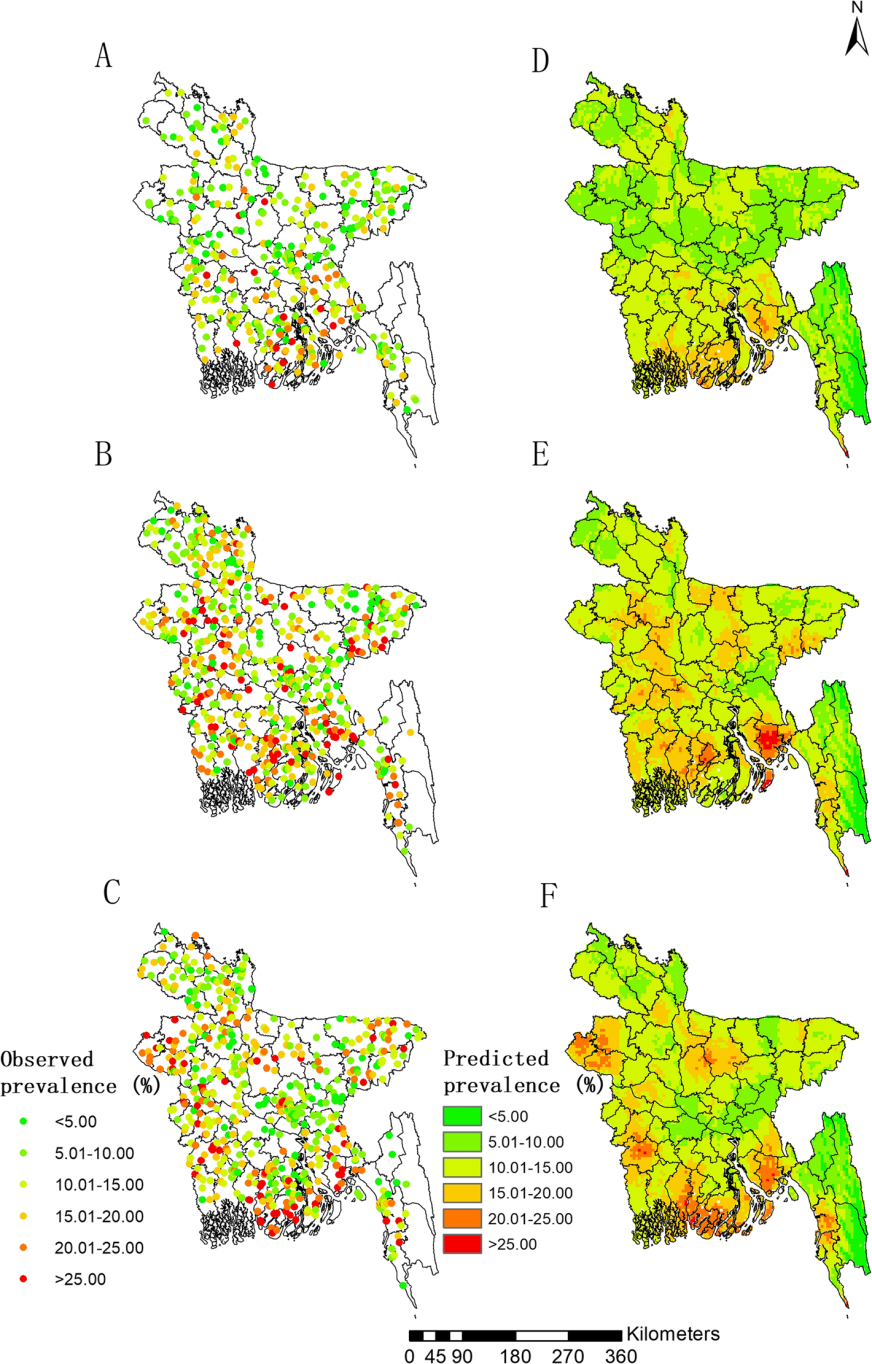
Observed prevalence and estimated risk maps of RTIs in Bangladesh in the three survey years. **a**, **b** and **c** present the survey locations and observed prevalence in the year 2007, 2011 and 2014, respectively; **d**, **e** and **f** present the model-based estimated risk maps based on the median of the posterior estimated distribution of infection risk for the year 2007, 2011, 2014, respectively

We included the surveys of 46,701 women (i.e., 10,996 in 2007, 17,842 in 2011 and 17,863 in 2014), aged 15–49 years old, who completed the DHS standard questionnaires, for the subsequent analysis. Summarized of the data resulted in a total of 1560 clusters, of which 361 in 2007, 600 in 2011 and 600 in 2014 (Fig. 1a, b and c, respectively). One cluster with 33 women surveyed in 2014 was not included in the geostatistical analysis due to the absence of coordinates. A summary of the surveys is shown in Table 1.

**Table 1:** Summary of DHS surveys in Bangladesh [34–36]

Indicators	Year			Total
	2007	2011	2014	
**Number of survey clusters**	361	600	600	1561
**Number of households interviewed (response rate %)**^**a**^	10,400 (99.4)	17,141 (97.9)	17,300 (98.5)	44,841 (98.5)
**Number of women aged 15–49 interviewed (response rate %)**^**b**^	10,996 (98.4)	17,842 (97.9)	17,863 (97.9)	46,701 (98.0)
**Raw prevalence of reported with RTI symptoms (%)**^**c**^	10.99	14.39	13.94	13.42
**Education years of women**^**d**^	4.52 ± 4.40	4.96 ± 4.06	5.30 ± 4.10	4.99 ± 4.17
**Education years of husbands**^**d**^	5.15 ± 5.01	5.39 ± 4.78	5.48 ± 4.79	5.37 ± 4.84
**BAS (%)**^**e**^	22.93	32.15	44.30	32.70
**Toilet improvement (%)**^**f**^	46.18	45.98	57.65	50.53
**Wealth index of household**^**g**^	−38,290 (−67,484;48,000)	−24,096 (−70,135;65,283)	−29,401 (−75,110;68,694)	−29,996 (− 70,774;64,069)

^a^ Calculated as households interviewed/households occupied

^b^ Respondents interviewed/eligible respondents

^c^ Calculated as overall number of positive/total number of interviewed

^d^ Expressed as mean ± standard deviation

^e^ The proportion of total live births that are attended by skilled health personnel (i.e., qualified doctor, nurse, midwife, family welfare visitor, or community skilled birth attendant)

^f^ The proportion of interviewed women that have improved toilet facility system (i.e., Flush/pour flush to piped sewer, septic tank, pit latrine, Ventilated improved pit (VIP) latrine, Pit latrine with slab)

^g^ Wealth index serves as an indicator of household-level wealth, constructed using household asset data via principal components analysis by DHS^28^, expressed as median (first quartile, third quartile)

### Geostatistical modeling and model validation

Based on the results of the variable selection (which are highly consistent regardless of whether DIC or MPL is used as the predictive performance measure, see Table S4 and Table S5 in revised Additional file 1), we selected the following variables for the final geostatistical logistic regression model: wealth index of household, elevation, human influence index (HII), land surface temperature (LST) in the daytime, normalized difference vegetation index (NDVI) and distance to the nearest fresh water body. Since the autoregressive coefficient is non-significant when defining temporal effect as AR1 process (Table S2 in Additional File 1), we set exchangeable time effect of the spatial-temporal process for the final model. The posterior summaries of the geostatistical model parameters are shown in Table 2. Negative associations were identified for the prevalence of reported having RTI symptoms with wealth index of household and elevation, while a positive association was found with distance to the nearest fresh water body, HII and NDVI.

**Table 2:** Posterior summaries (median and 95% BCI) of the geostatistical model parameters

Variables	Estimate
Wealth of household	−0.18 (− 0.23, − 0.13)^a^
Elevation	−0.10 (− 0.13, − 0.07)^a^
HII	0.08 (0.03, 0.12) ^a^
LST in the daytime (°C)	
< 26	0
26–28	−0.02 (− 0.10, 0.06)
> 28	−0.07 (− 0.23, 0.07)
Water distance	0.06 (0.03, 0.09)^a^
NDVI	0.06 (0.03, 0.10) ^a^
Spatial variance (${\sigma}_{sp}^2$)	0.15 (0.11, 0.20)
Range (*R*, km)	52.69 (36.45, 73.17)
Non-spatial variance **(**${\sigma}_{nonsp}^2\Big)$	0.06 (0.03, 0.10)

^a^Statistical significance

To be noted, all the autocorrelation coefficients of the predictor surfaces are greater than 0.9 (Table S6 in Additional file 1), suggesting high spatial smoothness of the corresponding surfaces, point extraction from which provide unbiased estimates. Thus, the coordinate displacement of DHS surveys had little impact on the geostatistical analysis.

10-fold cross model validation suggested that the Bayesian geostatistical logistic regression model was able to correctly estimate 74.49% of locations within a 95% BCI coverage. The mean error was 0.42%, suggesting that our models might have slightly underestimated the infection risk. According to the results of the sensitivity analysis, the model parameter estimates are almost the same before and after adjusting the smoothing parameter values (Table S3 in Additional file 1), indicating that the model is relatively stable.

### Estimated risk maps

Model-based estimated risk maps in 2007, 2011, 2014 are presented in Fig. 1d, e and f, respectively. The northern part of the country and southeastern portion of Chittagong Division show low (< 10%) to moderate (10–15%) prevalence while most southern areas of the country show moderate (10–15%) to high (15–20%) prevalence in 2007. Prevalence increased in the year 2011 in most areas of the country, which lead to a high prevalence (15–20%) in large areas of the western part and the southern coastal region, and a moderate prevalence (10–15%) in the majority of the remaining areas. To be noted, the prevalence in Noakhali District of the southeastern was estimated the highest (> 20%). The risk map in 2014 shows a more pronounced regional clustering. In addition to the southern region, the western Rajshahi Division, the middle of Khulna Division in the western part of the country, and the southwestern Chittagong Division were estimated to be very high-risk areas with prevalence estimated higher than 20%.

**Fig. 2:**
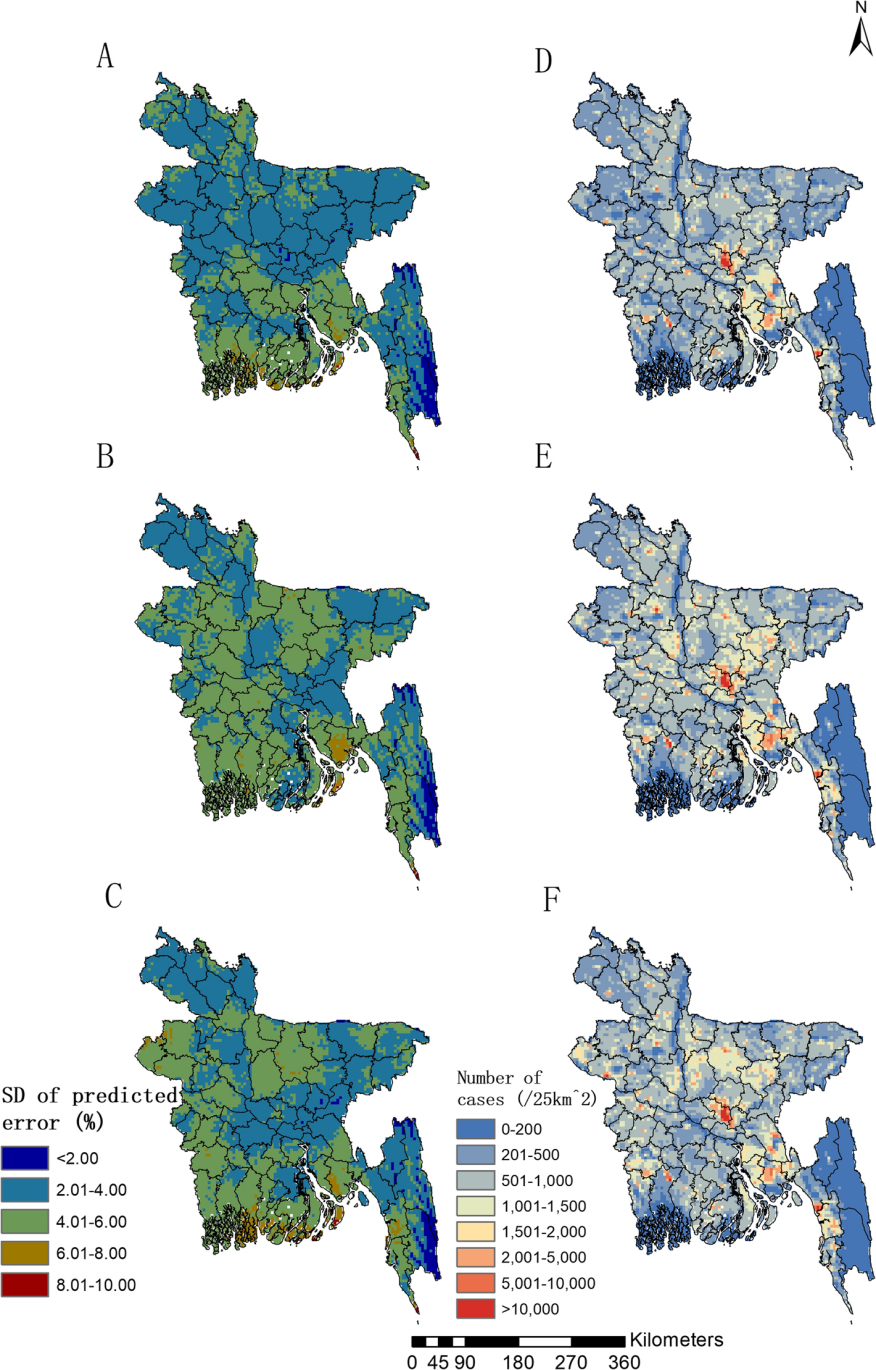
Estimated uncertainty and the number of women reported with RTI symptoms in Bangladesh. **a**, **b** and **c** present estimated uncertainty based on the standard deviation of the posterior estimated distribution of prevalence, for the year 2007, 2011 and 2014, respectively; (**d**), (**e**), (**f**) present the number of infected women of childbearing age based on the estimated prevalence and gridded population of the year 2007, 2011 and 2014, respectively

Figure 2a, b and c show the estimated uncertainty of prevalence for the year 2007, 2011 and 2014, respectively, which is generally low (< 6%) in most areas of the country. Risk maps for number of infected women aged 15–49 years old are present in Fig. 2d, e and f for the year 2007, 2011 and 2014, respectively. Large numbers of infected women (> 10,000/25km^2^) were estimated in areas with high population density, such as the capital city Dhaka in Dhaka Division and the second largest city Chittagong in Chittagong Division, while areas with very high infection risk, such as the areas of the western part, the southern coastal region and Noakhali District of the southeastern, mostly show moderate number of infected (500–5000/25km^2^).

### Estimates of population-adjusted prevalence and number of people infected

The nationwide population-adjusted predicted prevalence were estimated to be 11.11% (95% BCI: 10.50–11.68%), 13.93% (95% BCI: 13.34–14.54%) and 13.39 (95% BCI: 12.80–14.04%) in 2007, 2011 and 2014, respectively, which is comparable with the raw prevalence (Table 1 and Table 3). We estimated that 5.33 million (95% BCI: 5.10–5.59 million) women aged 15–49 reported with RTI symptoms in Bangladesh in 2014, significantly higher than that in 2007 (4.09 million, 95% BCI: 3.86–4.29 million), while similar as that in 2011 (5.36 million, 95% BCI: 5.13–5.59).

**Table 3:** Population-adjusted predicted prevalence (%) and estimated number of women (×10^3^) reported with RTI symptoms in Bangladesh^a^

Year	Division	WOCBA Population^b^	Prevalence(%)^c^	Estimated number^d^
**2007**	Barisal	1897.30	15.02 (13.22;16.81)	284.96 (250.73;318.86)
	Chittagong	6670.58	12.65 (11.15;13.96)	844.05 (743.54;931.49)
	Dhaka	9652.66	9.97 (8.82;11.29)	962.21 (851.09;1089.4)
	Khulna	4026.09	12.28 (10.68;13.92)	494.31 (430.19;560.54)
	Mymensingh	3087.49	10.78 (8.77;13.17)	332.68 (270.83;406.49)
	Rajshahi	4725.08	9.71 (8.22;11.34)	458.59 (388.18;535.61)
	Rangpur	3843.25	11.14 (9.46;13.2)	428.21 (363.38;507.24)
	Sylhet	2865.69	9.56 (8.27;11.09)	273.94 (237.06;317.78)
	**Total**	36,768.14	11.11 (10.50;11.68)	4086.54 (3858.99;4293.69)
**2011**	Barisal	1985.00	16.23 (14.59;17.95)	322.16 (289.69;356.35)
	Chittagong	6978.90	14.46 (13.28;15.85)	1009.29 (926.69;1105.96)
	Dhaka	10,098.81	12.84 (11.5;14.19)	1296.58 (1161.68;1432.92)
	Khulna	4212.18	15.58 (14.18;17.13)	656.19 (597.48;721.57)
	Mymensingh	3230.2	14.72 (12.63;17.16)	475.37 (408;554.31)
	Rajshahi	4943.48	14.81 (13.52;16.43)	732.22 (668.48;811.99)
	Rangpur	4020.88	12.09 (10.8;13.42)	486.07 (434.1;539.71)
	Sylhet	2998.14	12.61 (11.24;14.16)	377.95 (336.9;424.55)
	**Total**	38,467.59	13.93 (13.34;14.54)	5357.84 (5131.92;5594.92)
**2014**	Barisal	2056.02	16.52 (14.9;18.49)	339.69 (306.32;380.09)
	Chittagong	7228.59	13.78 (12.5;15.05)	996.22 (903.55;1088.09)
	Dhaka	10,460.13	11.36 (10.22;12.76)	1188.7 (1069.45;1334.2)
	Khulna	4362.88	15.77 (14.48;17.24)	687.95 (631.86;752.1)
	Mymensingh	3345.77	15.14 (12.81;17.72)	506.61 (428.48;592.91)
	Rajshahi	5120.35	14.65 (13.32;16.2)	750.27 (682.03;829.24)
	Rangpur	4164.74	11.53 (10.37;13.03)	480.08 (432.03;542.72)
	Sylhet	3105.41	12.12 (10.59;13.73)	376.51 (328.9;426.24)
	**Total**	39,843.89	13.39 (12.80;14.04)	5334.03 (5098.70;5593.01)

^a^ Calculations based on the median and 95% Bayesian credible interval

^b^ WOCBA:Women of childbearing age (i.e., aged 15–49 years)

^c^ Expressed as the predicted prevalence (95%Bayesian credible interval)

^d^ Expressed as the estimated number (95%Bayesian credible interval)

**Fig. 3:**
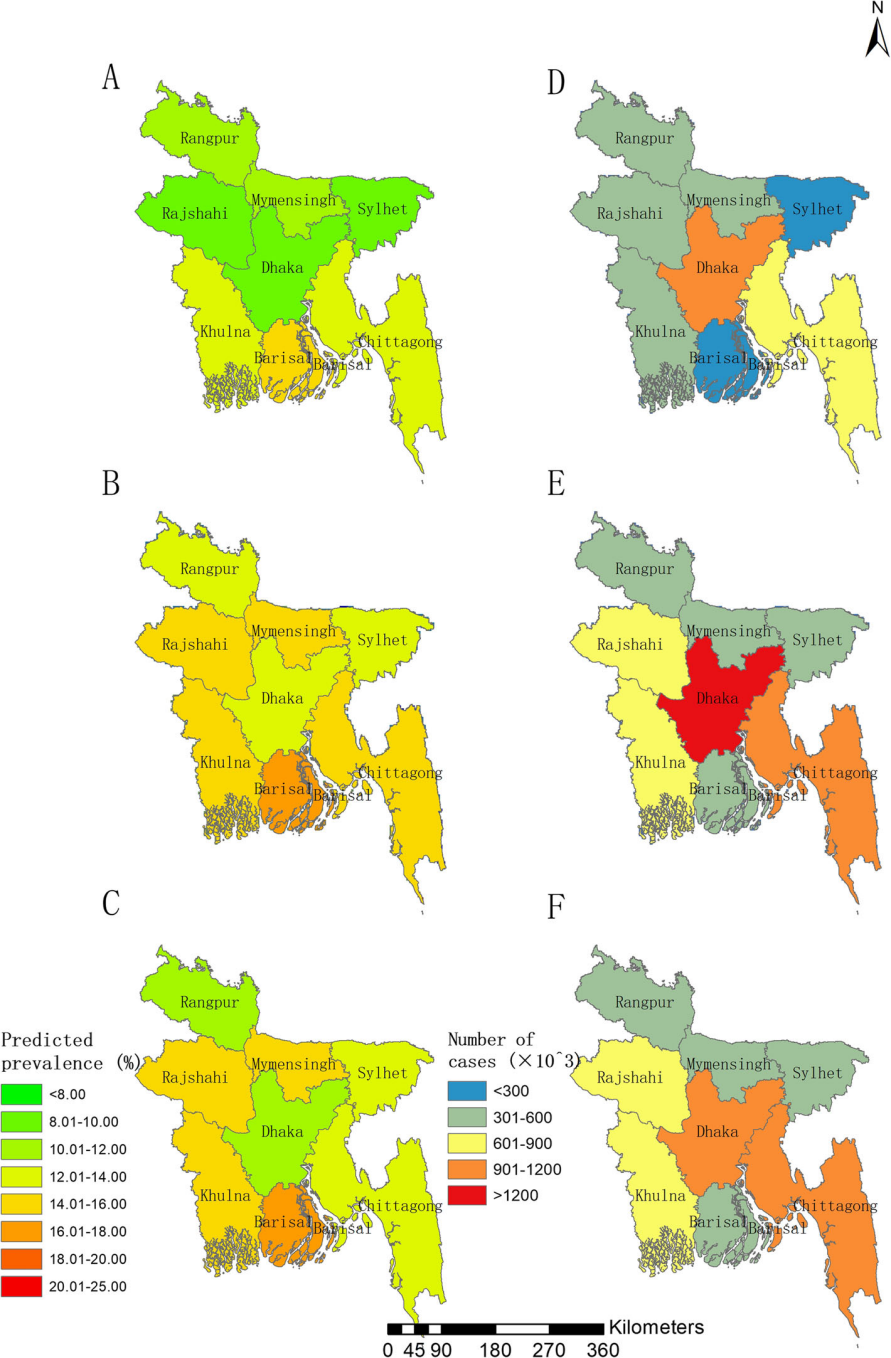
Estimated prevalence and number of women reported with RTI symptoms across Bangladesh at division level. **a**, **b** and **c** show the estimated prevalence in 2007, 2011, 2014, respectively, based on the median of the posterior estimated prevalence; **d**, **e** and **f** show the estimated number of infected women of childbearing age in 2007, 2011, 2014, respectively

**Fig. 4:**
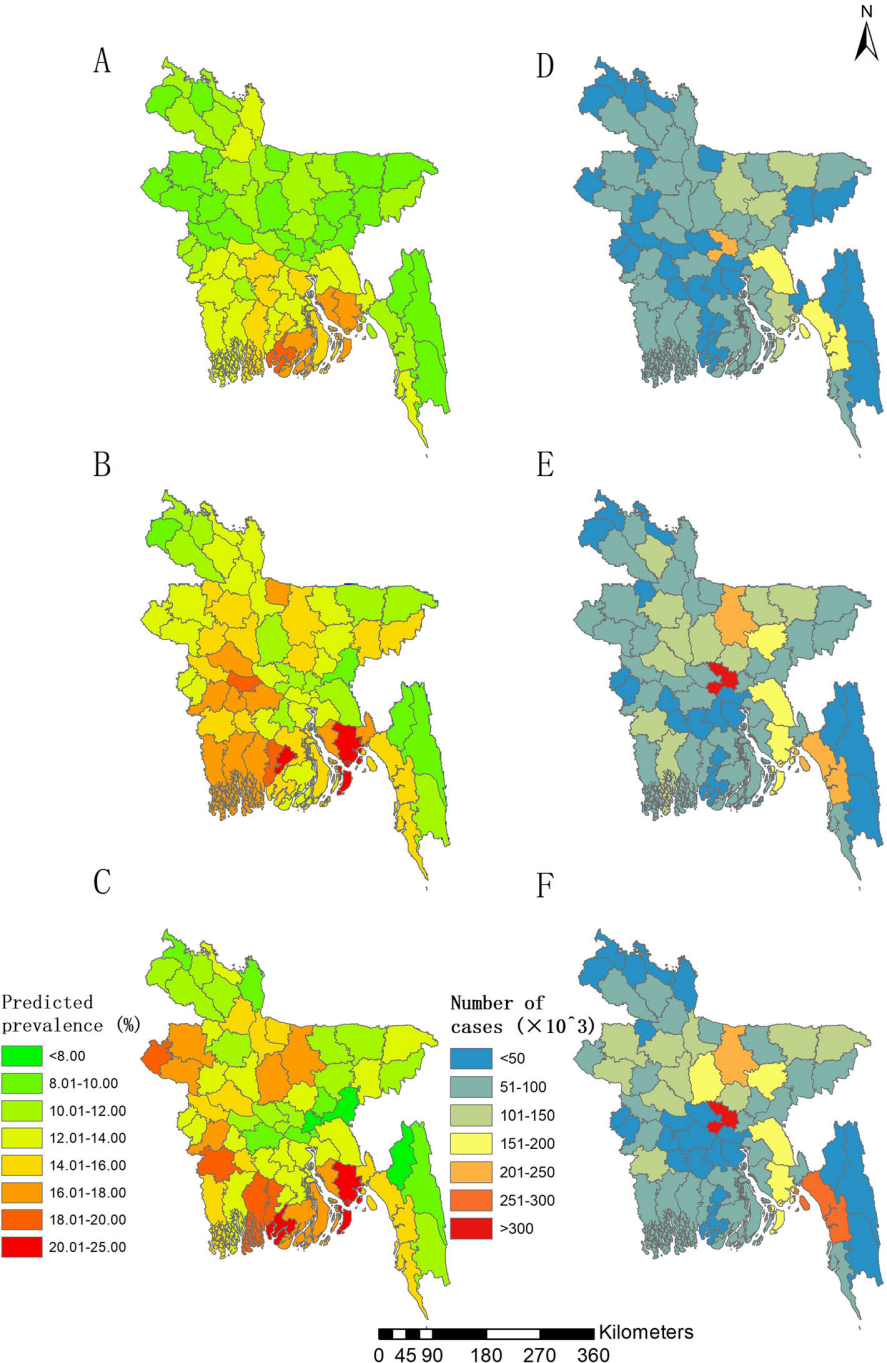
Estimated prevalence and number of women reported with RTI symptoms across Bangladesh at district level. **a**, **b** and **c** show the estimated prevalence in 2007, 2011, 2014, respectively, based on the median of the posterior estimated prevalence; **d**, **e** and **f** show the estimated number of infected women of childbearing age reported with RTI symptoms in 2007, 2011, 2014, respectively

The division Barisal shows the highest prevalence among all 8 divisions of Bangladesh in the three survey years, followed by divisions Khulna in the years 2011 and 2014. The division Dhaka had the highest number of infected, followed by Chittagong in the three surveys years (Table 3 and Fig. 3). From district-level perspective (Fig. 4 and Table S7 in Additional file 1), Barguna District, located in the eastern of the southern coastal region, had the highest infection prevalence among all 64 districts in 2014, that is 21.69% (95% BCI: 17.70–27.22%), followed by Noakali District (20.89, 95% BCI: 16.73–26.14%) and Nawabganj District (19.53, 95% BCI: 14.93–25.28%). The prevalence in Brahmanbaria District, located in the middle-eastern part, was estimated the lowest prevalence in 2007 and 2014 (i.e., 8,07, 7.29% in the year 2007 and 2014, respectively). Dhaka and Chittagong districts had the highest number of infected in all three survey years.

### Temporal changes

**Fig. 5:**
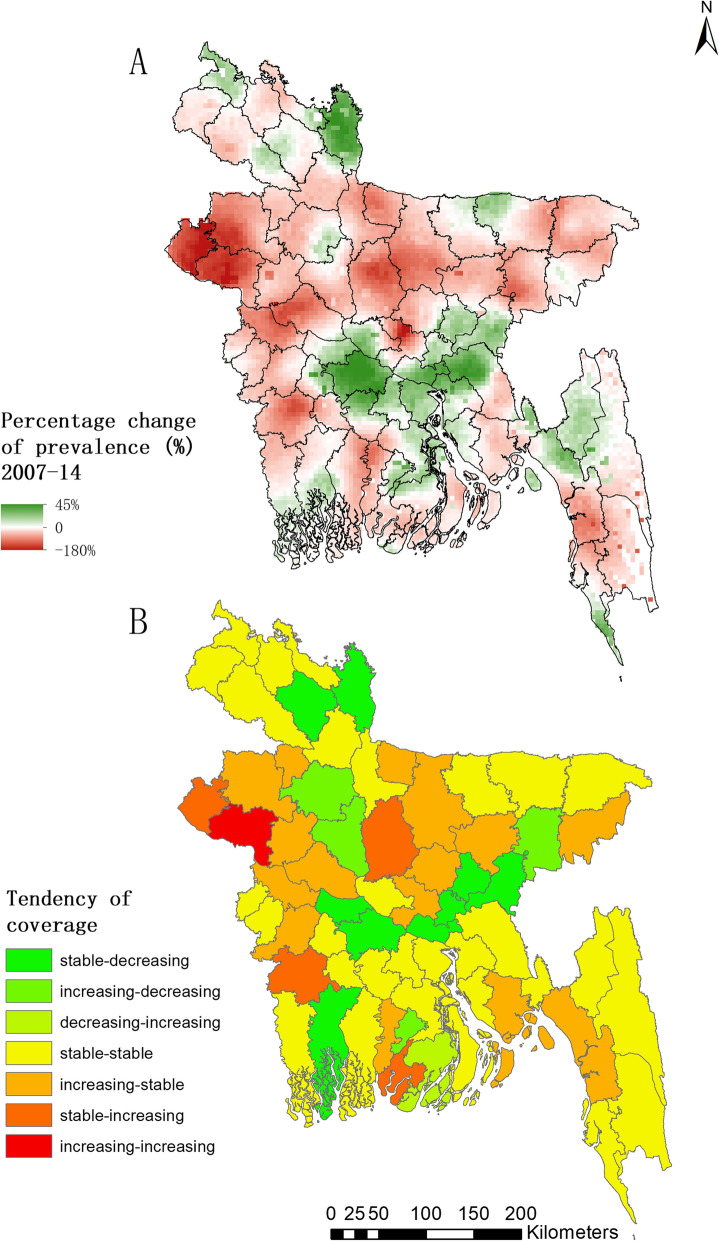
Temporal changes of RTI risk of women of childbearing age in Bangladesh. **a** depicts the pixel-level percentage change of RTI risk of women of childbearing age in Bangladesh from 2007 to 2014, calculated as the median estimated prevalence for 2007 minus that for 2014 and divided by that for 2007 in each grid; **b** presents the district-level temporal trends in the three survey years. The decreasing areas were defined as the districts with prevalence significantly decreased compared with the previous survey year (i.e., median of posterior estimated prevalence was less than the lower bound of 95% BCI of posterior estimated prevalence in the previous survey year). Similar definition was defined for the increasing areas. And the areas with predicted prevalence that did not significantly increase or decrease were considered as the stable areas

Pixel-level temporal relative change map in prevalence of self-reported RTI symptoms from 2007 to 2014 are shown in Fig. 5a. Considerable decrease of prevalence (dark green) only appeared in some areas of the northern, the middle and the middle-eastern parts of the country, while most areas show obvious increase of prevalence (from red to dark red), such as the western, the middle-northern and the southern parts of the country. Particularly, the highest increase, which is close to 180% compared to the prevalence in 2007, was located in the junction of three districts (Naogaon, Nawabganj, Rajshahi) in the northwestern Bangladesh. Temporal changes of prevalence during the three survey years at district level are present in Fig. 5b, where Rajshahi are the districts with prevalence increasing during all the survey years, and many districts in the middle-north show an increasing trend from 2007 to 2011 then become stable from 2011 to 2014. Only very few districts show a decreasing trend.

## Discussion

In this study, our goal is to better understand the problem of female RTIs in underdeveloped countries like Bangladesh and to inform pre-emptive and precise interventions. Through Bayesian spatiotemporal models, we have created high precision maps for 2007, 2011, and 2014. One advantage of our spatial-temporal model is that it is able to cope with the changing locations and quantity of sampled clusters in each of the surveys [37]. Moreover, spatial-temporal model fits all survey data in a single model, allowing more data to be utilized to improve the statistical efficiency.

Women in low- and middle-income countries (LMICs) tend not to seek health care unless they are suffering alarming RTI symptoms [9]. Lack of awareness and attention to RTIs has led to more serious consequences. For instance, one-third of preterm births are attributed to reproductive tract infections during pregnancy, which often go undetected and untreated in LMICs [38]. Large-scale implementation of effective interventions, such as screening and treatment cost waivers, is often difficult to achieve, especially in countries with limited resources. Therefore, it is very cost-effective to find the small geographical priority areas according to the high-precision map for intervention. We produced the first model-based, high-resolution risk maps of RTIs in the country, based on three national cross-sectional survey data of Bangladesh DHS. RTI risk maps at district- and division-levels were further summarized and presented, which are important for policy makers at different levels of administrative divisions.

Our population-adjusted estimates show that in Bangladesh, there were approximate 11.1% (95% BCI: 10.5–11.7%), 13.9% (95% BCI: 13.3–14.5%) and 13.4% (95% BCI: 12.8–14.0%) of women of childbearing age reported reproductive tract infections in 2007, 2011 and 2014, respectively. This risk may be underestimated, as definition of RTIs in our study is based on self-reported symptoms of having abnormal genital discharge or genital sore/ulcer, which are major indicators suggesting RTIs, rather than hospital confirmed diagnoses. The asymptomatic cases of RTIs are ignored [39]. On the other hand, women reported having abnormal genital discharge or genital sore/ulcer can be generally considered as in a more severe situation of RTIs than those with asymptomatic infections, thus our estimates reflect the cases with more serious RTIs that need to pay more attention to.

The high-resolution risk maps of RTIs not only identify priority areas but also show spatial details of temporal changes across survey years that assist spatial targeted intervention. The southern coastal areas, the western Rajshahi Division, the middle of Khulna Division in the western part of the country, and the southwestern Chittagong Division were estimated to be very high-risk areas (> 20%) in 2014 that urge attention. Most areas in the country show obvious increase of prevalence from 2007 to 2014, particularly, the junction of the three districts (Naogaon, Nawabganj, Rajshahi) in the northwestern Bangladesh, where special focus show be put. In addition, proper health resources are suggested to allocate preferentially not only to areas with high RTI risk but also to areas with large number of infected cases, such as high-population-density areas in the capital city Dhaka and the second largest city Chittagong.

A negative relationship was found between the risk of RTIs and the household wealth, which is consistent with previous studies [40–43]. This result supports the high RTI risk in the coastal areas of southern Bangladesh, which are among the poorest in the country. The income level may influence women’s health behaviors thus impact the transmission of pathogens. A study shows that women with high income were more likely to use contraceptives than those with low income [44]. In addition, women in the poor families were intend to have high-risk behaviors, such as earlier childbearing [45], earlier sexual debut [46, 47], and transactional sex [48]. We also identified a negative association for RTI risk with elevation and a positive one with distance to the nearest fresh water body. To our knowledge, there are no literatures ever reveal direct relationships of the above environmental factors with RTIs. However, these factors may influence the behaviors and socioeconomic status of people [49], thus show significant association with RTIs.

To obtain more accurate estimates of RTI risk, we made full use of available data from multiple sources, such as DHS, MODIS/Terra, WorldClim, SEDAC, SWBD and WorldPop (Table S1, Additional file 1). However, other potential influencing factors, such as social attitudes [50] and the number of sexual partners [51, 52], are not available at sub-national level, thus cannot be included in our analyses. In addition, as we did not obtain data at high spatial resolution for wealth of household, we used district-level data for risk prediction, by assuming levels of wealth within districts were similar, which may lead to spatial misalignment issue and result in bias. For WorldClim and SEDAC data, the data periods are before 2000 and corresponding updated data are unavailable, thus temporal misalignment may exist.

Nevertheless, our final model had reasonable predictive ability, as model validation suggests that it was able to correctly estimate 74.49% of locations within a 95% BCI coverage. However, the mean error, slightly larger than zero, may suggest a mild underestimate of the model. Considering omitted variable-bias (OVB) issue that results from the elimination of important variables a process where only one of the two highly correlated continuous variables is retained, we went back to the results and found that during this process, only one variable (i.e., education years of husbands) was dropped due to its highly correlated (with correlation coefficient 0.92) with the variable “education years of women”. From a professional point of view, the education of women themselves may influence the RTI more directly than that of their husbands, thus omitting this variable may cause little OVB.

Due to the unavailability of the newest Bangladesh DHS data in 2017, we analyzed the data from 2007 to 2014, which may not fully reflect the most recent situation. Nevertheless, most areas in Bangladesh show obvious increase of RTI risk from 2007 to 2011, then became stable between 2011 and 2014. The Bangladesh government has published a set of policies and strategies to improve women’s health since 2009, such as the National Adolescent Reproductive Health Strategy (2009), the National Education Policy (2010), the National Maternal Health Strategy (2011), the National Women’s Development Policy (2011) and the National Plan of Action on Adolescent Sexual and Reproductive Health (2013) [13]. These efforts may have certain effects on control of RTIs, as the infection risk in most areas of Bangladesh maintained stable instead of increase between 2011 and 2014. However, long-term evaluation is needed. Our analyses should be updated as soon as the 2017 DHS data is released. One the other hand, more efficient control interventions should be carried out in priority areas, which may include health education and hygiene, screening and empowering women. Effective health and personal hygiene education is thought an important measure for women RTI control, by improve women’s knowledge of RTIs, enhancing the awareness of the disease prevention and enabling them maintaining good life style [53]. Screening is another powerful method to detect asymptomatic infected women as early as possible [54]. Furthermore, other strategies such as empowering women, providing educational and employment opportunities for women and improving basic health care services, are advocated by WHO as essential practice for control of RTIs [55]. One successful example is the case in Daqing City of China, after a series of actions (e.g., reproductive health knowledge promotion, health file establishment and tracking), the prevalence of RTIs decreased significantly [56].

## Conclusion

In conclusion, we present the first model-based spatial-temporal risk estimates of RTIs at 5 × 5 km resolution in Bangladesh, which can aid control strategies targeted to priority areas cost-effectively. Our results show that more than one eighth of women of childbearing age reported symptoms suggesting RTIs in Bangladesh, urging the government to pay more attention to the worrying situation of female RTIs in the country.

## Supplementary Information

**Additional file 1.**

## Data Availability

The datasets supporting study are available in the following hyperlink: Demographic and Health Surveys (DHS), available at:http://dhsprogram.com/; Moderate Resolution Imaging Spectroradiometer (MODIS)/Terra, available at:http://modis.gsfc.nasa.gov/; WorldClim, available at: http://www.worldclim.org/current; Socioeconomic Data and Applications Center, available at: http://sedac.ciesin.org/; Shuttle Radar Topography Mission Water Body Data (SWBD), available at: http://gis.ess.washington.edu/data/vector/worldshore/index.html; World population, available at: www.worldpop.org.uk/data/data_sources/; World bank, available at: https://data.worldbank.org/country/bangladesh.

## Abbreviations

ADM1: Administrative divisions of level one
ADM2: Administrative divisions of level two
BAS: Births that are attended by skilled health personnel
BDHS: Bangladesh Demographic and Health Survey
BCI: Bayesian credible intervals
DHS: Demographic and Health Survey
DIC: Deviance Information Criterion
EAs: Enumeration areas
HII: Human influence index
HIV: Human immunodeficiency virus
LST: Land surface temperature
MODIS: Moderate Resolution Imaging Spectroradiometer
NDVI: Normalized difference vegetation index
RTIs: Reproductive tract infections
SEDAC: Socioeconomic Data and Applications Center
SWBD: Shuttle Radar Topography Mission Water Body Data
VIP: Ventilated improved pit
WOCBA: Women of childbearing age
WorldPop: World population
