# Supplementary material for: High-resolution mapping of reproductive tract infections among women of childbearing age in Bangladesh: a spatial-temporal analysis of the demographic and health survey
Source: BMC Public Health. 2021 Feb 12;21:342. doi: 10.1186/s12889-021-10360-4 (PMC7881647; doi:10.1186/s12889-021-10360-4)
Supplement: Supplementary file 1 — Additional file 1. [file 12889_2021_10360_MOESM1_ESM.docx]

## Additional file 1 - Supplemental materials

**Data sources**

**Table S1** Summary of data sources*****

| Variables | Data  Source | Data period | Temporal resolution | Spatial resolution |
| --- | --- | --- | --- | --- |
| Symptoms of RTIs, education of women, education of husbands, BAS^b^, number of children, toilet improvement,  wealth of household | DHS^a^ | 2007,  2011,  2014 | - | EA divided by DHS |
| NDVI^d^ | MODIS/Terra^c^ | 2000-2015 | 16 days | 1km |
| LST^e^ in the daytime,  LST^e^ at night | MODIS/Terra^c^ | 2000-2015 | 8 days | 1km |
| elevation | WorldClim^f^ | 2000 | - | 1km |
| moisture | WorldClim^f^ | 1950-2000 | - | 1km |
| HII^h^ | SEDAC^g^ | 1995-2004 | - | 1km |
| Urban extents | SEDAC^g^ | 1995-2004 | - | - |
| Water bodies | SWBD^i^ | 2000 | - | 30m |
| WOCBA^k^ | WorldPop^j^ | 2010 | - | 1km |
| Growth rate of population^l^ | World bank^m^ | 2007-2014 | - | - |

*Data were accessed during Jan 1 to Jan 15, 2019;

^a^ Demographic and Health Surveys (DHS), available at：http://dhsprogram.com/.

b Birth attendance by skilled provider

c Moderate Resolution Imaging Spectroradiometer (MODIS)/Terra, available at：http://modis.gsfc.nasa.gov/.

d Normalized difference vegetation index

e Land surface temperature (LST) day and night

f Available at: http://www.worldclim.org/current.

g Socioeconomic Data and Applications Center, available at: http://sedac.ciesin.org/.

h Human influence index

i Shuttle Radar Topography Mission Water Body Data (SWBD), available at: http://gis.ess.washington.edu/data/vector/worldshore/index.html.

j World population, available at: www.worldpop.org.uk/data/data_sources/.

k Women of childbearing age (age 15-49).

l To calibrate the population for 2007,2011,2014.

m Available at: https://data.worldbank.org/country/bangladesh.

**Statistical analysis**

**Calculating pixel-level number of infected women**

Pixel-level number of infectious women was calculated by multiplying the estimated prevalence by the number of women of childbearing age at the same pixel. The number of women of childbearing age in the three survey years is based on the pixel-level numbers in Bangladesh in 2010 obtained from WorldPop, adjusted by the corresponding population growth rates. The formula as follows:

$n_{t}=n_{0}\times exp[(t-2010)\times\frac{\alpha}{100}]$ (5)

Here, *t* represents the survey years 2007, 2011 and 2014. $n_{0}$ and $\alpha$ are the number of women of childbearing age in 2010 and population growth rate equaling to 1.2%^1^, respectively.

**Simultaneous** **autoregressive regression**

We fitted simultaneous autoregressive regression models, proposed by the guidelines of DHS, to test the smoothness of the predictor surfaces in our study. The function is as follows: $X=\eta WX+e$, where $W$ is neighborhood adjacency, row-standardized distance-based (100km) weight matrix: $W_{ij}=\{\begin{matrix} 0 if d_{i,j}>100km \\ \frac{1}{d_{i,j}} if d_{i,j}\leq100km \end{matrix}$ , where $d_{i,j}$ is the distance between observation $i$ and observation j. $X$ is dependent variable, $\eta$ is spatial autocorrelation coefficient, and $e$ is the error term (that is independent and identically distributed). Maximum likelihood estimation was used through the lagsarlm function in “R-spdep” package to inference $\eta$.

**Results**


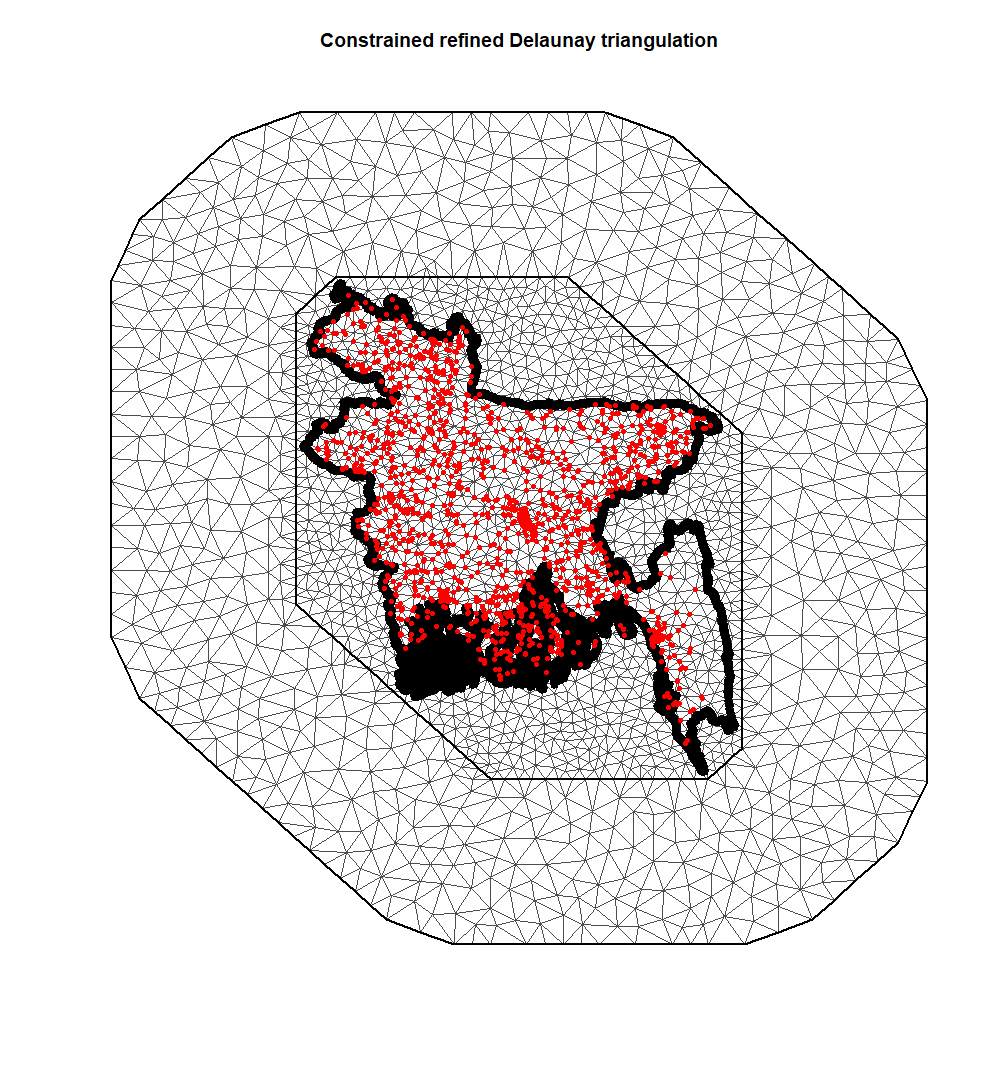


**Fig S1** Triangulation mesh used to obtain the spatial covariance, with the political boundary of Bangladesh in thick black and observation points in red

**Table S2** Posterior summaries (median and 95% BCI) of the geostatistical model parameters (defined temporal effect as AR1 process)

| **Variables** | **Estimate** |
| --- | --- |
| Wealth of household | -0.18 (-0.23, -0.13)* |
| Elevation | -0.10 (-0.13, -0.06)* |
| HII | 0.08 (0.03, 0.13)* |
| LST in the daytime (℃) |  |
| <26 | 0 |
| 26-28 | -0.0 2(-0.11, 0.071) |
| >28 | -0.08 (-0.23, 0.08) |
| Water distance | 0.06 (0.03 …0.09)* |
| NDVI | 0.06 (0.02, 0.09) |
| Spatial variance ($\sigma_{sp}^{2}$) | 0.14 (0.10, 0.20) |
| Range ($R$, km) | 65.02 (31.34, 68.83) |
| Non-spatial variance **(**$\sigma_{nonsp}^{2})$ | 0.080 (0.05, 0.12) |
| Autoregressive coefficient in AR1 ($\rho$) | 0.25 (-0.06,0.55) |

* Statistical significance

**Sensitivity analysis**

**Table S3** Posterior summaries (median and 95% BCI) of the geostatistical model parameters (smoothing parameter $\upsilon=0.5$)

| **Variables** | **Estimate** |
| --- | --- |
| Wealth of household | -0.18 (-0.23, -0.13)* |
| Elevation | -0.10 (-0.13, -0.07)* |
| HII | 0.08 (0.03, 0.12)* |
| LST in the daytime (℃) |  |
| <26 | 0 |
| 26-28 | -0.0 2(-0.10, 0.06) |
| >28 | -0.08 (-0.22, 0.07) |
| Water distance | 0.06 (0.03, 0.09)* |
| NDVI | 0.06 (0.02, 0.09) * |
| Spatial variance ($\sigma_{sp}^{2}$) | 0.14 (0.10, 0.20) |
| Range ($R$, km) | 54.62 (35.42, 80.04) |
| Non-spatial variance **(**$\sigma_{nonsp}^{2})$ | 0.17 (0.12, 0.22) |

* Statistical significance

**Variable selection**

**Table S4** Summary of variables functional form selection and their corresponding deviance information criterion (DIC) and marginal predictive likelihood (MPL)

| Variables | Functional form | DIC | MPL |
| --- | --- | --- | --- |
| education | linear form | 6672.980535 | 2.176043 |
|  | categorical form | 6674.82697 | 2.177305 |
| wealth | linear form | 6664.776638 | 2.172781 |
|  | categorical form | 6672.218321 | 2.176421 |
| children | linear form | 6687.203082 | 2.182222 |
|  | categorical form | 6689.397808 | 2.183489 |
| toilet improvement | linear form | 6683.050377 | 2.180890 |
|  | categorical form | 6685.165256 | 2.181704 |
| BAS | linear form | 6676.348124 | 2.177824 |
|  | categorical form | 6677.013112 | 2.178058 |
| elevation | linear form | 6687.436254 | 2.182312 |
|  | categorical form | 6690.292348 | 2.183808 |
| HII | linear form | 6686.612576 | 2.182178 |
|  | categorical form | 6688.733257 | 2.183319 |
| LST in the daytime | linear form | 6689.170321 | 2.183311 |
|  | categorical form | 6685.426699 | 2.181159 |
| LST at night | linear form | 6688.441696 | 2.182705 |
|  | categorical form | 6689.103827 | 2.182958 |
| water distance | linear form | 6685.331307 | 2.181200 |
|  | categorical form | 6686.754548 | 2.181522 |
| NDVI | linear form | 6688.635717 | 2.183078 |
|  | categorical form | 6689.520562 | 2.183705 |
| moisture | linear form | 6688.336648 | 2.183038 |
|  | categorical form | 6689.104087 | 2.183238 |

**Table S5** Summary of variables selection and their corresponding deviance information criterion (DIC) and marginal predictive likelihood (MPL)

| Number of variables | Combination of variables at minimum DIC value | DIC | Combination of variables at minimum MPL value | MPL |
| --- | --- | --- | --- | --- |
| 1 | wealth | 6664.754 | the same as the combination in DIC | 2.172796 |
| 2 | wealth+water distance | 6663.757 | the same as the combination in DIC | 2.171871 |
| 3 | wealth+HII+water distance | 6663.256 | wealth+LST in the daytime+water distance | 2.171494 |
| 4 | wealth+HII+LST in the daytime+water distance | 6662.339 | wealth+elevation+LST in the daytime+water distance | 2.171181 |
| 5 | wealth+elevation+HII+LST in the daytime+water distance | 6661.939 | the same as the combination in DIC | 2.170826 |
| 6 | **wealth+elevation+HII+LST in the daytime+water distance+NDVI** | **6661.887** | **the same as the combination in DIC** | **2.170679** |
| 7 | wealth+elevation+HII+LST in the daytime+water distance+NDVI+moisture | 6662.473 | the same as the combination in DIC | 2.171022 |
| 8 | wealth+children+elevation+HII+LST in the daytime+water distance+NDVI+moisture | 6663.54 | wealth+Toilet improvement+elevation+HII+LST in the daytime+water distance+NDVI+moisture | 2.171354 |
| 9 | wealth+children+elevation+HII+LST in the daytime+LST at night+water distance+NDVI+moisture | 6664.278 | wealth+Toilet improvement+elevation+HII+LST in the daytime+LST at night+water distance+NDVI+moisture | 2.171776 |
| 10 | wealth+children+BAS+elevation+HII+LST in the daytime+LST at night+water distance+NDVI+moisture | 6665.519 | wealth+Toilet improvement+BAS+elevation+HII+LST in the daytime+LST at night+water distance+NDVI+moisture | 2.172294 |
| 11 | wealth+children+Toilet improvement+BAS+elevation+HII+LST in the daytime+LST at night+water distance+NDVI+moisture | 6666.622 | the same as the combination in DIC | 2.172912 |
| 12 | education+wealth+children+Toilet improvement+BAS+elevation+HII+LST in the daytime+LST at night+water distance+NDVI+moisture | 6667.862 | the same as the combination in DIC | 2.173504 |
|  |  |  |  |  |

**Supplemental Tables**

**Table S6** The autocorrelation testing of ancillary data

| **Variable** | **The** **spatial autocorrelation coefficient** $\eta$ |
| --- | --- |
| Elevation | 0.9938 |
| HII | 0.9914 |
| Lst_day | 0.9932 |
| Lst_night | 0.9947 |
| NDVI | 0.9839 |
| Moisture | 0.9976 |
| population | 0.9395 |

**Table S7** Population-adjusted predicted prevalence (%) and estimated number of women (×10^3^) reported with RTIs symptoms in Bangladesh**^a^**

| **District** | **Year 2007** | | | **Year 2011** | | | | **Year 2014** | | |
| --- | --- | --- | --- | --- | --- | --- | --- | --- | --- | --- |
|  | **WOCBA**  **Population^b^** | **Prevalence(%)**^c^ | **Estimated number**^d^ | | **WOCBA**  **Population^b^** | **Prevalence(%)**^c^ | **Estimated number**^d^ | **WOCBA**  **Population^b^** | **Prevalence(%)**^c^ | **Estimated number**^d^ |
| **Bagerhat** | 364.54 | 14.37 (11.08;18.83) | 52.38 (40.4;68.63) | | 381.39 | 17.97 (14.28;22.43) | 68.54 (54.44;85.56) | 395.04 | 19.09 (15.07;23.12) | 75.4 (59.53;91.34) |
| **Bandarban** | 110.70 | 8.42 (5.86;11.97) | 9.32 (6.49;13.26) | | 115.82 | 11.5 (8.36;15.8) | 13.32 (9.69;18.3) | 119.96 | 11.17 (8.14;15.48) | 13.4 (9.76;18.57) |
| **Barguna** | 202.96 | 18.59 (13.97;23.53) | 37.74 (28.35;47.75) | | 212.34 | 15.62 (12.1;19.17) | 33.17 (25.69;40.71) | 219.94 | 21.69 (17.70;27.22) | 47.71 (38.93;59.86) |
| **Barisal** | 507.84 | 12.9 (10.68;15.72) | 65.5 (54.24;79.86) | | 531.31 | 15.2 (12.62;17.71) | 80.77 (67.04;94.1) | 550.32 | 12.68 (10.58;15.26) | 69.78 (58.22;83.99) |
| **Bhola** | 411.90 | 14.41 (11.63;18.1) | 59.34 (47.91;74.57) | | 430.93 | 15.62 (12.81;18.88) | 67.32 (55.2;81.35) | 446.35 | 16.61 (13.23;20.3) | 74.15 (59.07;90.63) |
| **Bogra** | 874.46 | 10.06 (7.25;13.81) | 87.95 (63.41;120.79) | | 914.88 | 15.76 (13.05;19.38) | 144.19 (119.39;177.29) | 947.61 | 11.73 (9.52;14.62) | 111.19 (90.23;138.5) |
| **Brahamanbaria** | 776.44 | 8.09 (5.85;11.47) | 62.79 (45.38;89.07) | | 812.33 | 9.87 (7.53;12.64) | 80.16 (61.15;102.69) | 841.39 | 7.29 (5.53;9.44) | 61.37 (46.49;79.45) |
| **Chandpur** | 594.64 | 13.85 (10.73;17.79) | 82.38 (63.83;105.79) | | 622.13 | 13.41 (10.28;17.28) | 83.4 (63.95;107.51) | 644.39 | 12.48 (9.77;16.06) | 80.4 (62.95;103.5) |
| **Chittagong** | 1675.31 | 10.83 (8.78;13.23) | 181.44 (147.17;221.71) | | 1752.74 | 14.13 (11.92;16.87) | 247.61 (208.89;295.71) | 1815.45 | 14.35 (12.03;17.06) | 260.54 (218.4;309.8) |
| **Chuadanga** | 299.39 | 10.85 (8.19;14.88) | 32.47 (24.51;44.56) | | 313.23 | 12.41 (9.32;16.15) | 38.88 (29.18;50.57) | 324.44 | 13.68 (10.62;17.57) | 44.37 (34.44;57.01) |
| **Comilla** | 1283.06 | 13.95 (11.33;17.19) | 178.99 (145.35;220.62) | | 1342.37 | 11.41 (9.22;13.95) | 153.19 (123.77;187.29) | 1390.39 | 13.83 (11.18;16.83) | 192.27 (155.44;233.94) |
| **Cox's Bazar** | 458.15 | 13.26 (9.61;18.38) | 60.76 (44.03;84.22) | | 479.33 | 15.64 (11.99;21.03) | 74.97 (57.47;100.8) | 496.48 | 14.94 (11.36;19.34) | 74.17 (56.38;96) |
| **Dhaka** | 2961.67 | 8.19 (6.52;10.49) | 242.66 (193.19;310.72) | | 3098.56 | 11.53 (9.38;13.91) | 357.24 (290.72;430.89) | 3209.42 | 10.9 (8.91;13.25) | 349.74 (286.05;425.29) |
| **Dinajpur** | 753.14 | 10.34 (7.68;13.92) | 77.85 (57.82;104.83) | | 787.95 | 11.2 (9.13;13.48) | 88.27 (71.93;106.23) | 816.14 | 11.4 (9.37;14.33) | 93.02 (76.44;116.97) |
| **Faridpur** | 467.56 | 14.34 (10.16;19.38) | 67.04 (47.53;90.62) | | 489.17 | 16.25 (12.23;21.8) | 79.5 (59.85;106.65) | 506.67 | 9.3 (6.69;12.89) | 47.14 (33.91;65.29) |
| **Feni** | 340.01 | 13.25 (9.89;17.8) | 45.06 (33.62;60.52) | | 355.73 | 17.56 (12.83;22.97) | 62.48 (45.63;81.73) | 368.45 | 14.09 (10.61;18.5) | 51.91 (39.1;68.17) |
| **Gaibandha** | 581.34 | 12.33 (8.93;16.34) | 71.65 (51.92;95) | | 608.21 | 12.45 (10.14;15.12) | 75.73 (61.64;91.93) | 629.97 | 14.98 (12.24;18.45) | 94.38 (77.1;116.25) |
| **Gazipur** | 860.17 | 10.03 (7.42;13.44) | 86.24 (63.79;115.62) | | 899.93 | 15.71 (12.18;20.33) | 141.41 (109.59;182.99) | 932.13 | 12.56 (9.98;15.75) | 117.12 (92.99;146.82) |
| **Gopalganj** | 304.76 | 14.11 (10.3;19.16) | 42.99 (31.38;58.39) | | 318.84 | 13.79 (10.2;18.09) | 43.96 (32.51;57.69) | 330.25 | 12.91 (9.57;17.21) | 42.63 (31.59;56.82) |
| **Habiganj** | 583.28 | 8.29 (6.49;10.51) | 48.33 (37.84;61.31) | | 610.24 | 15.63 (12.65;19.31) | 95.36 (77.22;117.81) | 632.08 | 12.02 (9.67;14.8) | 76 (61.15;93.56) |
| **Jamalpur** | 621.70 | 11.11 (7.77;15.28) | 69.05 (48.32;95.02) | | 650.43 | 13.68 (10.29;17.57) | 88.98 (66.95;114.25) | 673.71 | 15.34 (11.91;20.32) | 103.38 (80.23;136.88) |
| **Jessore** | 710.70 | 12.31 (9.58;15.8) | 87.49 (68.1;112.32) | | 743.55 | 15 (12.17;17.89) | 111.55 (90.49;133.04) | 770.15 | 18.31 (15.15;22.06) | 140.98 (116.66;169.87) |
| **Jhalokati** | 156.44 | 14.47 (10.87;18.44) | 22.63 (17.01;28.85) | | 163.67 | 21.57 (18.02;26.61) | 35.3 (29.49;43.55) | 169.52 | 13.7 (10.92;17.8) | 23.22 (18.51;30.17) |
| **Jhenaidah** | 471.69 | 12.77 (10.26;16.18) | 60.23 (48.38;76.32) | | 493.50 | 16.24 (13.48;20.24) | 80.15 (66.54;99.89) | 511.15 | 16.52 (13.48;19.99) | 84.44 (68.92;102.16) |
| **Joypurhat** | 253.35 | 9.96 (6.65;14.3) | 25.23 (16.85;36.23) | | 265.06 | 15.17 (11.05;20.71) | 40.21 (29.29;54.88) | 274.54 | 12.96 (9.49;17.14) | 35.58 (26.06;47.06) |
| **Khagrachhari** | 150.49 | 8.29 (4.69;13.5) | 12.48 (7.06;20.32) | | 157.45 | 9.71 (6.07;15.03) | 15.28 (9.56;23.66) | 163.08 | 7.36 (4.48;12.3) | 12 (7.3;20.06) |
| **Khulna** | 591.10 | 12.87 (9.98;16.63) | 76.09 (58.99;98.31) | | 618.43 | 16.48 (13.62;20.14) | 101.93 (84.24;124.53) | 640.55 | 13.53 (10.9;16.79) | 86.67 (69.8;107.54) |
| **Kishoreganj** | 1269.47 | 8.7 (6.24;11.97) | 110.5 (79.26;152) | | 1328.14 | 13.17 (9.74;17.04) | 174.96 (129.34;226.28) | 1375.66 | 11.8 (8.8;15.71) | 162.39 (121.12;216.18) |
| **Kurigram** | 471.37 | 13.79 (9.74;17.6) | 64.99 (45.92;82.98) | | 493.16 | 13.72 (10.92;17.11) | 67.68 (53.87;84.4) | 510.80 | 9.75 (7.59;12.31) | 49.8 (38.78;62.89) |
| **Kushtia** | 501.18 | 9.08 (6.68;12.22) | 45.53 (33.47;61.27) | | 524.34 | 14.15 (11.21;17.49) | 74.18 (58.78;91.72) | 543.10 | 15.38 (12.36;18.85) | 83.55 (67.11;102.37) |
| **Lakshmipur** | 447.81 | 16.36 (12.16;21.76) | 73.28 (54.47;97.45) | | 468.51 | 17.63 (13.94;21.78) | 82.59 (65.33;102.04) | 485.27 | 16.07 (12.29;20.6) | 77.97 (59.65;99.96) |
| **Lalmonirhat** | 300.87 | 11.86 (8.86;16.3) | 35.68 (26.65;49.06) | | 314.77 | 12.4 (9.58;15.42) | 39.03 (30.15;48.53) | 326.04 | 12.46 (9.55;16.05) | 40.63 (31.15;52.33) |
| **Madaripur** | 294.83 | 13.61 (10.15;19.01) | 40.13 (29.93;56.05) | | 308.45 | 11.99 (8.97;16.02) | 36.99 (27.67;49.4) | 319.49 | 12.28 (8.71;17.76) | 39.24 (27.84;56.75) |
| **Magura** | 246.35 | 13.6 (9.85;18.3) | 33.51 (24.28;45.07) | | 257.74 | 16.64 (12.55;21.79) | 42.89 (32.33;56.16) | 266.96 | 12.86 (9.5;16.63) | 34.34 (25.37;44.39) |
| **Manikganj** | 372.80 | 10.02 (6.7;14.08) | 37.34 (24.98;52.5) | | 390.03 | 13.89 (10.17;18.24) | 54.18 (39.66;71.16) | 403.98 | 10.42 (7.74;14.41) | 42.08 (31.27;58.2) |
| **Maulvibazar** | 446.72 | 10.38 (7.85;13.3) | 46.36 (35.08;59.43) | | 467.36 | 14.31 (11.57;17.99) | 66.88 (54.08;84.07) | 484.08 | 11.98 (9.54;15.06) | 57.99 (46.16;72.88) |
| **Meherpur** | 178.21 | 11.37 (7.34;17.68) | 20.27 (13.08;31.51) | | 186.45 | 13.19 (9.49;18.26) | 24.58 (17.69;34.04) | 193.12 | 13.75 (9.98;18.48) | 26.56 (19.27;35.69) |
| **Munshiganj** | 352.97 | 10.96 (8.02;14.5) | 38.67 (28.29;51.18) | | 369.28 | 12.42 (9.18;16.42) | 45.87 (33.91;60.62) | 382.49 | 8.61 (5.87;11.56) | 32.93 (22.43;44.21) |
| **Mymensingh** | 1339.63 | 11.11 (8.24;14.7) | 148.84 (110.35;196.87) | | 1401.55 | 15.47 (12.65;18.77) | 216.79 (177.23;263.05) | 1451.69 | 16.95 (13.88;20.74) | 246.05 (201.54;301.08) |
| **Naogaon** | 658.59 | 9.8 (7.33;13.23) | 64.55 (48.28;87.11) | | 689.03 | 13.87 (10.96;16.94) | 95.54 (75.5;116.73) | 713.69 | 16.06 (13.12;19.76) | 114.63 (93.65;140.99) |
| **Narail** | 186.78 | 10.82 (7.6;14.95) | 20.21 (14.19;27.92) | | 195.42 | 14.84 (10.75;20.23) | 29.01 (21.01;39.52) | 202.41 | 14.04 (10.39;18.97) | 28.41 (21.04;38.4) |
| **Narayanganj** | 703.25 | 8.58 (6.5;11.71) | 60.3 (45.69;82.38) | | 735.76 | 10.75 (8.16;13.75) | 79.07 (60.05;101.17) | 762.08 | 7.62 (5.54;10.05) | 58.06 (42.2;76.61) |
| **Narsingdi** | 572.36 | 9.38 (6.51;13.01) | 53.68 (37.28;74.47) | | 598.82 | 12.6 (9.46;16.59) | 75.42 (56.67;99.34) | 620.24 | 8.38 (6.19;11.26) | 52.01 (38.39;69.82) |
| **Natore** | 430.05 | 9.51 (6.88;13.86) | 40.89 (29.57;59.59) | | 449.93 | 14.21 (11.1;18.03) | 63.92 (49.96;81.13) | 466.02 | 13.4 (10.02;17.13) | 62.43 (46.71;79.84) |
| **Nawabganj** | 413.10 | 9.03 (5.75;14.01) | 37.29 (23.77;57.87) | | 432.19 | 13.52 (10.46;18.08) | 58.42 (45.19;78.16) | 447.66 | 19.53 (14.93;25.28) | 87.44 (66.85;113.15) |
| **Netrakona** | 785.89 | 10.48 (7.86;14.44) | 82.34 (61.76;113.51) | | 822.21 | 13.19 (9.86;17.91) | 108.46 (81.07;147.23) | 851.63 | 11.71 (8.19;15.76) | 99.72 (69.74;134.2) |
| **Nilphamari** | 432.26 | 9.27 (6.38;13.56) | 40.06 (27.58;58.62) | | 452.24 | 11.61 (8.96;14.94) | 52.51 (40.53;67.57) | 468.42 | 10.44 (7.98;13.66) | 48.92 (37.38;63.97) |
| **Noakhali** | 681.33 | 17.42 (13.2;22.01) | 118.67 (89.93;149.93) | | 712.82 | 24.71 (20.52;29.61) | 176.14 (146.24;211.06) | 738.32 | 20.89 (16.73;26.15) | 154.26 (123.56;193.06) |
| **Pabna** | 655.78 | 9.66 (7.02;12.76) | 63.32 (46.05;83.67) | | 686.09 | 16.57 (13.21;20.12) | 113.65 (90.6;138.04) | 710.63 | 14.54 (11.54;18.27) | 103.33 (82.01;129.86) |
| **Panchagarh** | 234.73 | 10.53 (7.13;16.1) | 24.72 (16.74;37.79) | | 245.58 | 10 (7.11;13.69) | 24.56 (17.46;33.62) | 254.37 | 9.53 (6.81;13) | 24.25 (17.31;33.07) |
| **Patuakhali** | 347.10 | 17.88 (14.37;21.87) | 62.07 (49.88;75.91) | | 363.15 | 13.9 (11.32;17.04) | 50.47 (41.1;61.87) | 376.14 | 17.9 (15.28;21.34) | 67.34 (57.48;80.29) |
| **Pirojpur** | 271.07 | 13.34 (10.46;16.97) | 36.16 (28.34;46) | | 283.60 | 19.16 (15.8;23.08) | 54.32 (44.82;65.46) | 293.74 | 19.01 (15.87;22.88) | 55.84 (46.61;67.19) |
| **Rajbari** | 271.51 | 13.19 (9.23;18.93) | 35.82 (25.06;51.41) | | 284.06 | 18.2 (13.53;23.27) | 51.7 (38.44;66.1) | 294.23 | 11.99 (8.76;15.77) | 35.28 (25.79;46.39) |
| **Rajshahi** | 652.02 | 8.55 (6.11;12.05) | 55.78 (39.85;78.55) | | 682.15 | 12.62 (10.28;15.34) | 86.09 (70.13;104.63) | 706.56 | 17.37 (14.67;20.45) | 122.74 (103.65;144.51) |
| **Rangamati** | 152.63 | 8.14 (5.57;11.81) | 12.43 (8.5;18.03) | | 159.68 | 9.79 (6.94;13.47) | 15.64 (11.08;21.51) | 165.39 | 8.6 (5.57;12.6) | 14.22 (9.21;20.85) |
| **Rangpur** | 730.41 | 10.76 (7.94;14.37) | 78.62 (58;104.97) | | 764.17 | 13.23 (11.02;16.33) | 101.11 (84.2;124.82) | 791.51 | 10.85 (8.77;13.3) | 85.84 (69.41;105.26) |
| **Satkhira** | 476.13 | 12.82 (9.39;17.23) | 61.02 (44.72;82.03) | | 498.14 | 16.12 (12.92;20.63) | 80.3 (64.37;102.78) | 515.96 | 15.68 (12.75;20) | 80.91 (65.8;103.19) |
| **Shariatpur** | 284.67 | 14.69 (10.27;21.13) | 41.81 (29.22;60.16) | | 297.82 | 12.51 (9.08;16.66) | 37.27 (27.04;49.6) | 308.48 | 12.25 (8.52;17.73) | 37.79 (26.27;54.7) |
| **Sherpur** | 340.27 | 9.03 (6.11;13.19) | 30.74 (20.8;44.89) | | 356.00 | 16.4 (11.89;21.86) | 58.37 (42.32;77.82) | 368.74 | 14.38 (10.07;20.54) | 53.04 (37.14;75.74) |
| **Sirajganj** | 787.74 | 10.15 (7.28;13.99) | 79.97 (57.38;110.17) | | 824.15 | 15.61 (12.78;18.92) | 128.66 (105.34;155.94) | 853.63 | 12.7 (10.38;15.83) | 108.38 (88.64;135.1) |
| **Sunamganj** | 1015.33 | 9.85 (7.76;12.53) | 100.01 (78.76;127.27) | | 1062.26 | 10.75 (8.4;13.71) | 114.24 (89.24;145.62) | 1100.27 | 11.39 (9.09;15.09) | 125.32 (99.99;166) |
| **Sylhet** | 820.36 | 9.56 (7.54;12.03) | 78.46 (61.86;98.7) | | 858.28 | 11.61 (9.56;13.74) | 99.64 (82.03;117.91) | 888.98 | 12.82 (10.69;15.16) | 113.97 (95.03;134.77) |
| **Tangail** | 936.65 | 9.96 (7.5;13.66) | 93.31 (70.23;127.92) | | 979.94 | 11.5 (8.91;14.55) | 112.69 (87.27;142.53) | 1015.00 | 16.1 (12.77;21.48) | 163.42 (129.61;218.04) |
| **Thakurgaon** | 339.13 | 9.81 (6.32;15.57) | 33.26 (21.42;52.8) | | 354.80 | 9.37 (6.73;12.79) | 33.25 (23.89;45.38) | 367.50 | 11.07 (8.34;14.55) | 40.68 (30.64;53.46) |
| **Total** | 36768.14 | 11.11 (10.50;11.68) | 4086.54(3858.99;4293.69) | | 38467.59 | 13.93 (13.34;14.54) | 5357.84 (5131.92;5594.92) | 39843.89 | 13.39 (12.80;14.04) | 5334.03(5098.70;5593.01) |

^a^ Calculations based on the median and 95% Bayesian credible interval

b WOCBA：Women of childbearing age (i.e., aged 15-49 years)

^c^ Expressed as the predicted prevalence (95%Bayesian credible interval)

^d^ Expressed as the estimated number (95%Bayesian credible interval)

**Reference**

1 The world bank group.2019. https://data.worldbank.org/country/bangladesh. Accessed 25 April 2019.
